# Predictive value of the TyG index and rheumatoid factor for cardiovascular disease risk in a rheumatoid arthritis population: data from a survey of 418 patients

**DOI:** 10.1186/s12944-022-01735-6

**Published:** 2022-11-17

**Authors:** Zihan Wang, Tianyi Lan, Liubo Zhang, Jing Luo, Jinping Wang, Lin Li, Qingwen Tao

**Affiliations:** 1 Beijing University of Chinese Medicine, Beijing, People’s Republic of China; 2 Traditional Chinese Medicine Department of Rheumatism, China-Japan Friendship Hospital, Beijing, People’s Republic of China; 3 Beijing Key Laboratory of Immune Inflammatory Disease, Beijing, People’s Republic of China; 4 Department of Integrative Cardiology, China-Japan Friendship Hospital, Beijing, People’s Republic of China

**Keywords:** Rheumatoid arthritis, Cardiovascular disease, TyG index, Rheumatoid factor, Clinical prediction model

## Abstract

**Objective:**

To investigate the correlation between the triglyceride-glucose (TyG) index and rheumatoid factor levels and the existence of cardiovascular disease in patients in the rheumatoid arthritis population and to analyze their potential value in predicting the risk of cardiovascular disease.

**Methods:**

Patients with rheumatoid arthritis treated by the Traditional Chinese Medicine Department of Rheumatism of the China-Japan Friendship Hospital from 2019–01 to 2021–12 were included in this retrospective study. Regression analysis was performed with multifactor-corrected multimodal logistic models to observe the correlation between the TyG index and rheumatoid factor and cardiovascular disease risk, construct predictive models and assess the potential predictive value of the variables on cardiovascular disease risk with receiver operating characteristic curves. The results were further corrected by sensitivity analysis and trend tests.

**Results:**

A total of 418 patients with rheumatoid arthritis were included in the study. In the rheumatoid arthritis population, high rheumatoid factor (OR = 1.002, 95% CI = 1.001–1.002, *P* < 0.001), high TyG index (OR = 1.057, 95% CI = 1.008–1.109, *P* = 0.022), advanced age (OR = 1.080, 95% CI = 1.050–1.112, *P* < 0.001), and low physical activity (OR = 2.848, 95% CI = 1.195–6.785, *P* = 0.018) were independent risk factors for the existence of cardiovascular disease in patients. The combined coefficient calculated on the basis of the TyG index and rheumatoid factor was used to plot the receiver operating characteristic curve with an area under the curve of 0.791, which can be used to predict the potential risk of cardiovascular disease in patients with rheumatoid arthritis. Further sensitivity analysis found that the marker of focus remained associated with cardiovascular disease risk in a high-physical activity population with rheumatoid arthritis. The final trend test found a linear trend between the TyG index, rheumatoid factor levels and the risk of cardiovascular disease.

**Conclusion:**

In the rheumatoid arthritis population, the TyG index and rheumatoid factor have some potential predictive value in determining the risk of cardiovascular disease, and the predictive efficacy is better when the two tests are combined.

## Introduction

Epidemiological surveys have shown that the prevalence of rheumatoid arthritis (RA) has reached 0.5–1% [1] and is one of the most common multisystem autoimmune diseases, with immune-mediated chronic inflammation as its main pathological feature [2]. Chronic synovial inflammation is the main manifestation of RA, and the disease can be secondary to a variety of organ functional impairments. Cardiovascular disease (CVD) is a group of diseases that seriously endanger human health security [3]. The four categories of hypertension, coronary heart disease (CHD), arrhythmia, and heart failure (HF) are typical representatives of CVD, in the pathogenesis of which the inflammatory response plays an important role, directly affecting myocardial cell necrosis and intercellular electrical signaling and accelerating the development of lesions [4]. RA and CVD share a common inflammatory response mechanism, and it is now believed that RA patients have an additional 50% risk of developing CVD compared to the normal population [5]. Meanwhile, the role of immunomodulatory mechanisms has been increasingly emphasized in recent years, and the "immune-inflammatory" signaling pathway plays an important role in the development of CVD [6]. Rheumatoid factors (RF) are considered one of the important serological criteria for the diagnosis of RA [7]. The triglyceride-glucose (TyG) index is a novel index for the evaluation of glucolipid metabolism levels [8], which can be easily and conveniently applied in clinical work, and recent studies have found an association between an elevated TyG index and cardiac or vascular changes such as atherosclerosis [9, 10]. Based on this, we hope to clarify whether the TyG index combined with RF has clinical predictive power for CVD risk in the specific context of high immune-inflammatory activation in RA patients, with a view to helping clinicians identify people at high risk of CVD in RA patients and provide an optimal direction for the treatment process.

## Materials and methods

### Selection methods

This is a retrospective study based on a population with RA. The population with RA who visited the Traditional Chinese Medicine Department of Rheumatism of the China-Japan Friendship Hospital from 2019–01 to 2021–12 at the last visit was enrolled, in which patients aged 18–75 years with retrievable complete case information were included. Patients with a combination of other rheumatological diseases, diabetes, liver or renal insufficiency, acute infection, presence of tumors, severe hematopoietic disorders, and factors affecting data collection due to psychiatric language were excluded. Patients who used triglyceride-lowering drugs (fibrates, niacin or omega-3) or glucose-lowering agents or drugs related to CVD (NSAIDs, corticosteroids, antimalarials, etc.) were also excluded from the study. The study was registered with the Chinese Clinical Trials Registry (No. ChiCTR2200057350). The study was approved by the Clinical Research Ethics Committee of the China-Japan Friendship Hospital (No. 2020–133-K86), and our study was conducted in accordance with the Declaration of Helsinki. Each patient signed an informed consent form at the time of treatment, allowing the use of clinical records for further clinical studies.

### Diagnosis criteria

The diagnosis of RA was made with reference to the ACR/EULAR criteria published in 2010 [11], using the item scores determined by the guidelines. CVD includes the four main categories of CHD, essential hypertension, arrhythmias (including various types of arrhythmic conditions such as supraventricular, ventricular or bradyarrhythmia), and HF, which are diagnosed by experienced clinicians according to their respective international classification diagnostic standards [12–15].

### Research methods

#### Basic information

The basic information of the study population was included, such as sex, age, body mass index (BMI), smoking and alcohol consumption, daily physical activity (low or high physical activity was classified based on 60 min of physical activity of moderate intensity or more per day), and the main symptoms of the patients were recorded.

#### Physical and chemical examination

All patients had venous blood specimens collected on an empty stomach in the early morning of the 2nd day after admission and tested for natriuretic peptide (BNP), homocysteine (Hcy), fasting glucose, triglycerides (TG), low-density lipoprotein cholesterol (LDL-C), blood uric acid (Ua), high-sensitivity C-reactive protein (Hs-CRP), and RA-related serological indicators such as C-reactive protein (CRP), erythrocyte sedimentation rate (ESR), rheumatoid factor (RF), anti-cyclic citrullinated polypeptide antibody (anti-CCP antibody), immunoglobulin IgG, complement C3, and complement C4 at the Department of Laboratory Medicine, China-Japan Friendship Hospital. The TyG index is based on the formula [10], TyG index = Ln [triglycerides (mg/dL) * fasting glucose (mg/dL)/2].

The corrected QT interval (QTc) was obtained by recording the electrocardiogram during hospitalization, and then echocardiography was carried out by echocardiographers who measured common parameters, including 2D ultrasound, left ventricular end-diastolic diameter (LVIDd), left ventricular posterior wall thickness (PWTd), and septal thickness (SWTd), according to the DEVEREUX-corrected ventricular mass formula [16]: LVM (g) = 0.8 × {1.04 [(LVIDd + PWTd + SWTd)^3^- LVIDd ^3^]} + 0.6 g, and the body surface area formula: BSA (m^2^) = 0.0061 × height (cm) + 0.0128 × weight (kg)—0.1529, calculating the left ventricular mass index (LVMI) = LVM/BSA.

#### Outcome events

According to the available medical records, the combination of CVD (including new or previously diagnosed CHD, hypertension, arrhythmia, and HF) was recorded as the primary outcome event. The diagnosis of each type of CVD was based on the respective disease criteria and was determined by a senior clinician.

#### Sample size calculation

The preestimation of 10–15 variables, including "TyG index" and "RF", might be associated with the existence of CVD in RA patients, with a minimum sample size of approximately 100–150 patients with CVD to follow the principle of approximately 10 outcome events per variable in regression analysis [17]. The final study included 418 patients with RA, including 173 patients with the combined existence of CVD.

### Statistical processing

Statistical analysis and visualization were performed in the R Programming Language (Version 4.1.1). Normally distributed data are expressed as the mean ± standard deviation, nonnormal distributions are expressed as the median and quartiles, and count data are expressed as percentages. When comparing the sample values of two groups, the independent samples t test was used for normally distributed data, the rank sum (Wilcoxon) test was used for nonnormally distributed data, and the chi-square test was used for count data.

In the regression analysis, the correlation between the outcome variable "CVD" and the predictor variable was analyzed. A multivariable-adjusted regression model was developed to focus on whether the TyG index and RF levels speculate the existence of CVD. According to the type of covariates, model 1 was adjusted for age and gender, based on which model 2 was adjusted for smoking, alcohol consumption and physical activity. Model 3 was further adjusted for continuous variables such as "TyG index" and "RF" and used as the main model. To evaluate the predictive ability of the TyG index combined with RF for CVD risk in RA patients by plotting the receiver operating characteristic curve (ROC), reporting the area under the curve (AUC), and comparing the AUC differences of the curves related to each index by the Delong method. The coordinates of each point on the ROC were observed, the maximum value of AUC was taken to obtain the best cutoff point, and the corresponding sensitivity and specificity were also obtained. Youden's index was calculated to indicate the model veracity, and consistency analysis was performed to demonstrate Cohen's kappa coefficient. Further sensitivity analyses were performed, and possible correlations between the target variables and CVD were corrected by stratification tests. Finally, a trend test was added. For the statistical process, *P* < 0.05 was considered statistically significant.

## Results

### Basic clinical information of the population

**Fig. 1:**
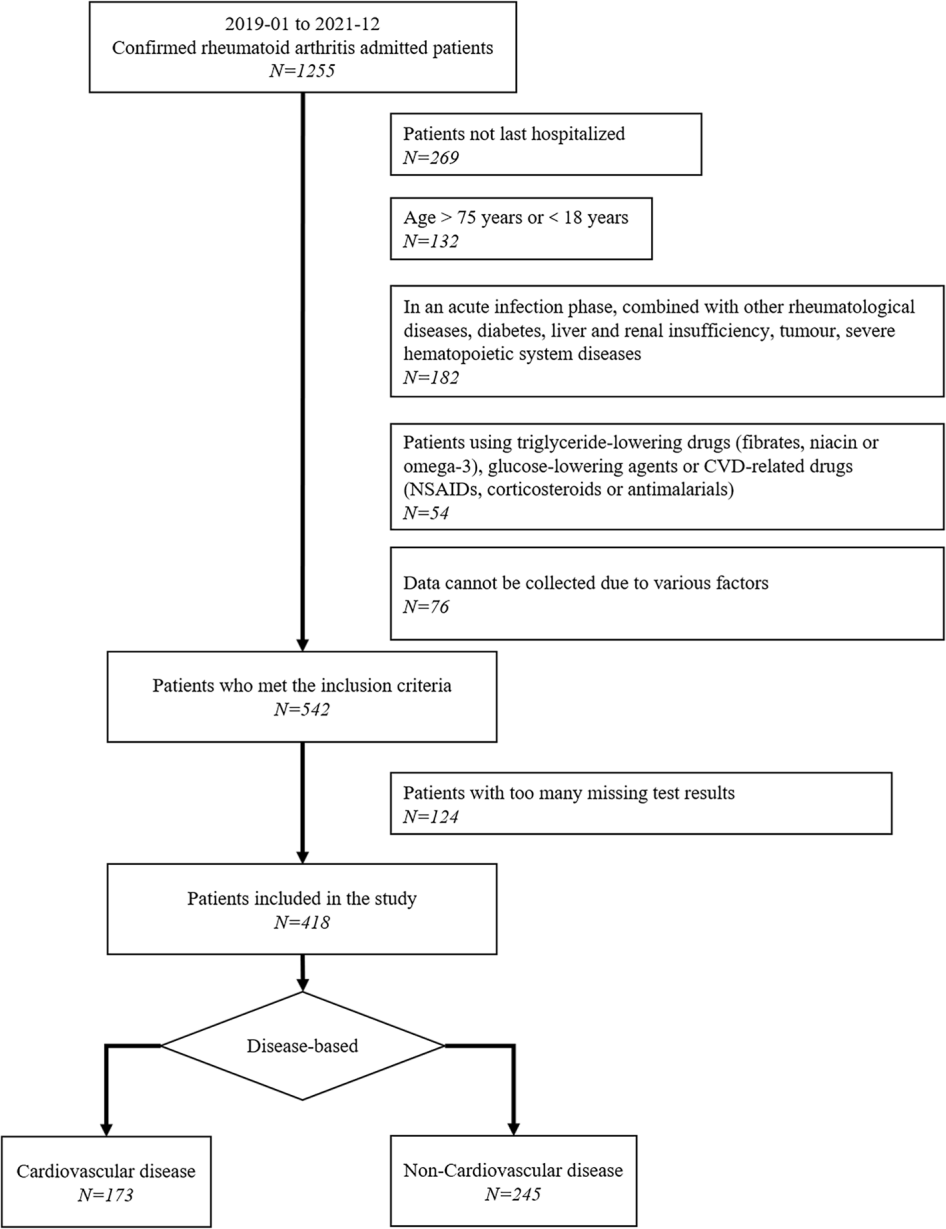
Flow chart of patient inclusion

The study included 418 patients with RA, and the specific inclusion process is shown in Fig. 1.

We analyzed the basic information of the RA population included in the study, as shown in Table 1. In the two groups of patients with combined CVD and without CVD, the proportions of sex, smoking and alcohol consumption were more consistent, and the test results showed similar levels of ESR, CRP, anti-CCP antibody, immunoglobulin IgG, complement C3, complement C4, Hs-CRP, and LDL-C, none of which were significantly different. We found that in the RA population, patients who developed CVD were older, had a higher BMI, had a higher proportion of low-physical activity, and had a longer duration of RA compared to those without CVD, with higher levels of TyG index, RF, and Hcy in the test, and the differences in these data were statistically significant (all *P* < 0.05). Patients with CVD did not show significant changes in QTc interval and BNP levels but possessed a higher LVMI, which we consider may be due to a much larger proportion of hypertensive patients with a correspondingly enlarged cardiac performance.

**Table 1:** Basic clinical information of the RA population

	Total(*N* = 418)	CVD(*N* = 173)	Non-CVD(*N* = 245)	*P* values^△^
Sex [male, N (%)]	106 (25.4)	48 (27.7)	58 (23.7)	0.363
Age (years)	54.44 ± 13.09	60.44 ± 9.88	50.20 ± 13.43	** < 0.001**
BMI (Kg/m^2^)	23.22 ± 3.86	23.87 ± 4.45	22.76 ± 3.31	**0.004**
CVD to Classify [N (%)]				
CHD	40 (9.6)	40 (23.1)	0	
Hypertension	126 (30.1)	126 (72.8)	0	
Arrhythmia	61 (14.6)	61 (35.3)	0	
HF	4 (1.0)	4 (2.3)	0	
Low-physical activity [N (%)]	61 (14.6)	44 (25.4)	17 (6.9)	** < 0.001**
Alcohol consumption [N (%)]	10 (2.4)	7 (4.0)	3 (1.2)	0.100
Somking [N (%)]	72 (17.2)	35 (20.2)	37 (15.1)	0.171
Duration of RA (years)	5.00 (1.00,10.00)	6.00 (1.50,15.00)	4.00 (1.00,10.00)	**0.011**
TyG index	8.40 ± 0.63	8.48 ± 0.59	8.34 ± 0.66	**0.030**
RF (IU/mL)	69.50(20.00,214.50)	88.10(20.00,431.25)	59.00(20.00,166.50)	**0.007**
ESR (mm/h)	35.22 ± 28.26	35.19 ± 28.58	35.24 ± 28.10	0.987
CRP (mg/dL)	1.04(0.39,2.67)	1.02(0.37,2.48)	1.04(0.39,2.74)	0.997
Anti-CCP Antibody (U/mL)	472.00(25.00,1491.00)	567.00(0,2274.00)	383.00(32.50.1283.50)	0.189
Immunoglobulin IgG (mg/dL)	1367.55 ± 411.41	1346.54 ± 436.07	1382.35 ± 393.42	0.394
Complement C3 (mg/dL)	94.16 ± 21.33	93.34 ± 20.27	94.74 ± 22.08	0.522
Complement C4 (mg/dL)	20.40 ± 6.73	20.37 ± 6.49	20.43 ± 6.91	0.923
Hs-CRP (mg/L)	8.34(2.67,22.87)	8.29(2.70,21.15)	8.65(2.58,22.91)	0.897
Hcy (μmol/L)	12.75 ± 6.76	13.86 ± 8.79	11.86 ± 4.37	**0.010**
LDL-C (mmol/L)	2.68 ± 0.77	2.65 ± 0.79	2.71 ± 0.75	0.474
Ua (μmol/L)	268.93 ± 86.17	280.94 ± 93.39	260.42 ± 79.78	**0.017**
QTc Interval (ms)	427.30 ± 26.86	428.51 ± 30.74	426.43 ± 23.75	0.447
LVMI (g/m^2^)	79.68 ± 22.05	84.68 ± 24.34	74.01 ± 17.62	**0.001**
BNP (pg/mL)	43.95(22.80,80.18)	53.20(28.50,112.60)	40.60(19.10,67.00)	0.054

*Abbreviations: CVD* cardiovascular disease, *BMI* body mass index, *CHD* coronary atherosclerotic heart disease, *HF* heart failure, *RF* rheumatoid factor, *ESR* erythrocyte sedimentation rate, *CRP* C-reactive protein, *Hs-CRP* hypersensitive C-reactive protein, *Hcy* homocysteine, *LDL-C* low-density lipoprotein cholesterol, *UA* uric acid, *LVMI* left ventricular mass index, *BNP* B-type natriuretic peptide

^△^Differential analysis of the data between the CVD and non-CVD groups, with bolded values representing statistical significance

### Logistic regression of relevant predictor variables

All variables with *P* < 0.2 after differential comparison in the results Sect. (Basic clinical information of the population section) were selected for regression analysis as a way to further investigate the correlation between the TyG index and RF levels and the risk of CVD in patients with RA. To control for the interaction between covariates, a multivariate adjusted regression model was constructed, as shown in Table 2. In model 1, which was adjusted for age and sex, the results indicated that higher age was associated with the existence of CVD (OR = 1.078, 95% CI = 1.057–1.101, *P* < 0.001). Model 2 was further adjusted for smoking, alcohol consumption and physical activity. The results found that, in addition to the effect of advanced age, the likelihood of CVD was higher in the low-physical activity population (OR = 2.888, 95% CI = 1.541–5.413, *P* < 0.001). After final correction using model 3, the results showed that in the RA population, four categories of factors, RF (OR = 1.001, 95% CI = 1.001–1.002, *P* < 0.001), TyG index (OR = 1.057, 95% CI = 1.008–1.109, *P* = 0.022), age (OR = 1.080, 95% CI = 1.050- 1.112, *P* < 0.001), and low-physical activity (OR = 2.845, 95% CI = 1.193–6.785, *P* = 0.018) were associated with the risk of CVD. Using the final model 3 as the main model, the model equation Logit(P) = Ln[P/(1-P)] = -6.323 + 0.056* TyG Index + 0.002* RF + 0.077* Age + 1.047* low-physical activity was constructed. The model was tested by an Omnibus test *P* < 0.001, -2 log likelihood value = 289.62, adjusted R-square = 0.37, Hosmer‒Lemeshow test *P* = 0.259 (> 0.05). The model was generally plausible, the fit was good, and the results obtained were statistically significant.

**Table 2:** CVD risk-related multimodel regression analysis with multivariate adjustment

	Beta	OR (95% *CI)*	*P* values^△^
Model 1^a^			
Constant	-4.564		
Age (years)	0.076	1.078 (1.057–1.101)	** < 0.001**
Model 2^b^			
Constant	-4.353		
Age (years)	0.069	1.071 (1.050–1.094)	** < 0.001**
Low-physical activity [N (%)] ^d^	1.061	2.888 (1.541–5.413)	** < 0.001**
Model 3^c^			
Constant	-6.323		
Age (years)	0.077	1.080 (1.050–1.112)	** < 0.001**
Low-physical activity [N (%)] ^d^	1.047	2.848 (1.195–6.785)	**0.018**
TyG Index	0.056	1.057 (1.008–1.109)	**0.022**
RF (IU/mL)	0.002	1.002 (1.001–1.002)	** < 0.001**

The ^△^Bolded value represents statistical significance

^a^Mode l was adjusted according to age and sex

^b^Model 2 was further adjusted for physical activity, alcohol consumption and smoking

^c^Model 3 was based on Model 2. The continuous variables "TyG index", "RF","BMI", "duration of RA", "anti-CCP antibody", "Hcy" and "Ua" included in Table 1 were further adjusted and used as the main model

^d^Compared to the study cohort with high physical activity

### Potential predictive value of the TyG index combined with RF for CVD risk

**Fig. 2:**
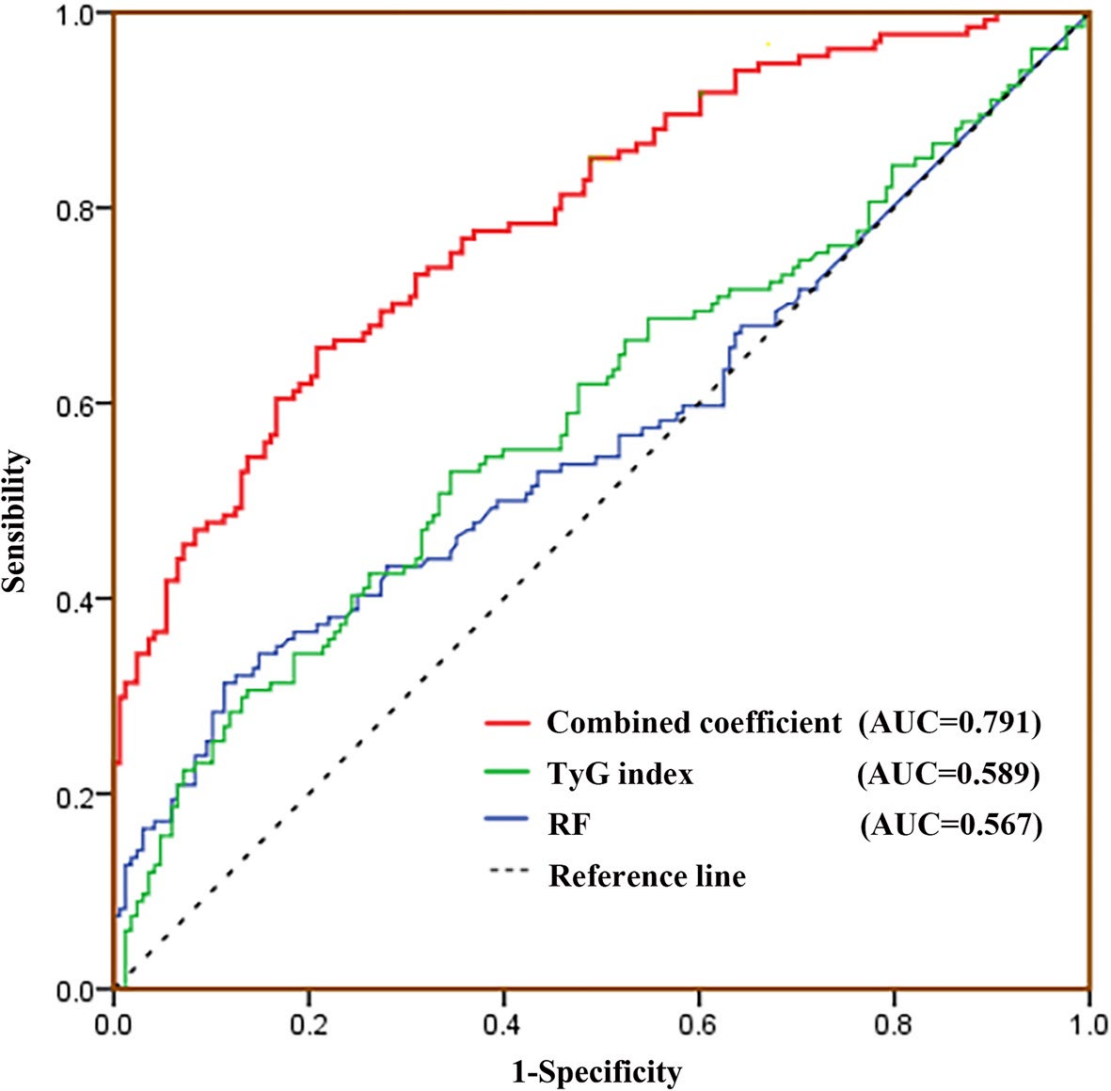
ROC curves for the “CVD”. Red line: Combined coefficient, AUC = 0.791, 95% CI = 0.740–0.841. Green line: TyG index, AUC = 0.589, 95% CI = 0.524–0.655. Blue line: RF, AUC = 0.567, 95% CI = 0.501–0.634. Dotted line: Reference line

We further analyzed the predictive power of the target variables for CVD risk. The combined coefficient Logit(P) was obtained according to the displayed model 3, and the positive diagnosis was based on the existence of "CVD" status. The ROC was plotted using the TyG index, RF and combined coefficient together, and the AUC of each curve was reported. The results are shown in Fig. 2. The combined coefficient had the largest area under the curve (AUC = 0.791, 95% CI = 0.740–0.841), followed by the TyG index (AUC = 0.589, 95% CI = 0.524–0.655) and RF (AUC = 0.567, 95% CI = 0.501–0.634). The combined coefficient corresponded to a larger AUC (*P* < 0.001) than the use of the TyG index or RF alone, and the difference was statistically significant.

The optimal cutoff point was obtained according to the ROC, and the model could predict the "CVD" status of RA patients when the TyG index was ≥ 8.42, RF was ≥ 249 IU/mL or the combined coefficient was ≥ 0.502. The combined coefficient was used to predict the negative predictive value = 79.17% and positive predictive value = 65.67% in the sample population. The ROC plotted with the combined coefficient finally calculated Youden index = 0.448, which represents its higher veracity and can better predict the risk of CVD in RA patients. After testing the model's predictions for consistency, Cohen's kappa coefficient = 0.446 (*P* = 0.004) was calculated, suggesting that this method of predicting CVD risk has moderately strong consistency with the original diagnostic criteria. The accuracy of the prediction is evaluated as shown in Table 3.

**Table 3:** Optimal cutoff points for indicators and their predictive coefficients for CVD risk

	Cutoff	Sensitivity (%)	Specificity (%)	Youden Index	Cohen’s Kappa Coefficient
TyG index	8.42	51.22	63.88	0.151	0.146
RF	249	31.98	86.78	0.186	0.197
Combined coefficient	0.502	65.67	79.17	0.448	0.446

### Sensitivity Analysis

Even in the non-RA population, age and physical activity are widely identified as factors associated with CVD [18]. Therefore, we performed further sensitivity analyses. Patients with RA were stratified according to age (mean age of the population in the study was 54 years) and physical activity (low or high), and independent variables were reported with TyG index and RF as the main observed variables. Table 4 shows the results of the sensitivity analysis based on model 3.

**Table 4:** Sensitivity analysis of CVD risk in patients with RA^b^

	CHD	TyG Index		RF (IU/mL)	
	(*N* = 173)	OR (95% *CI*)	*P* values^△^	OR (95% *CI*)	*P* values^△^
Age^a^					
54 years (*N* = 225)	127	1.054 (0.995–1.116)	0.074	1.002 (1.001–1.003)	**0.007**
≤ 54 years (*N* = 193)	46	1.079 (1.002–1.161)	**0.043**	1.002 (1.001–1.003)	**0.006**
Physical activity					
Low (*N* = 61)	44	1.123 (0.941–1.340)	0.197	1.002 (0.997–1.009)	0.309
High (*N* = 357)	129	1.053 (1.001–1.109)	**0.050**	1.002(1.001–1.002)	**0.001**

The ^△^Bolded value represents statistical significance

^a^The average age of the RA patient population was used as the cutoff value, average age = 54 years

^b^The regression model is based on Model 3

Overall, the final results did not change substantially across analyses, and correlation analyses of the TyG index and RF with CVD continued to find associations consistent with the main model after adjusting for age and physical activity. However, no significant correlation was observed between the TyG index and the risk of CVD in the relatively advanced age (*P* = 0.074) populations. Most likely because of the small sample size, statistical associations between the TyG index (*P* = 0.197), RF (*P* = 0.309) and CVD were also not observed in the low-physical-activity population.

### Trendiness test

Previous results found a statistical correlation between the TyG index, RF and CVD risk in the overall RA population. We further calculated the quartiles of the TyG index and quartiles of RF, divided the intervals and took the median value of each group (Q1-4), and thus carried out the trend test (Table 5). The highest range of the target variable was at Q4, and using this as a control and relying on regression analysis, it was found that RA patients with lower levels of TyG index (*P*_Trend_ = 0.026) and RF (*P*_Trend_ < 0.001) had a trend toward lower CVD existence.

**Table 5:** Trend test on CVD risk in the RA population

	TyG Index	Median	OR(95% *CI*)	RF (IU/mL)	Median	OR(95% *CI*)
Q1	≤ 8.05	7.83	0.586 (0.333–1.031)	≤ 20.00	20.00	0.345(0.195–0.613)
Q2	8.06–8.35	8.18	0.418 (0.234–0.747)	20.01- 69.50	33.50	0.386(0.215–0.693)
Q3	8.36–8.69	8.48	0.547 (0.309–0.966)	69.51–214.50	121.00	0.461(0.266–0.797)
Q4	> 8.69	8.97	1.000^a^	> 214.50	522.00	1.000^a^
*P* Values for Trend^△^			**0.026**			** < 0.001**

The ^△^Bolded value represents statistical significance

^a^In the regression analysis, the Q4 range was used as a control

## Discussion

Patients with RA are chronically exposed to high levels of "immune-inflammatory" activation [19], and they have a higher risk of CVD than the normal population due to the effects of disturbed immune and inflammatory factors. The TyG index is a conversion value calculated based on TG and glucose, which to some extent reflects the body's glucolipid metabolism. It includes TG and glucose, two blood indicators that are closely related to CVD and has been increasingly used in cardiovascular research in recent years. RF is one of the commonly used autoantibodies in the evaluation of rheumatic immune-related diseases, and its elevation represents the immune activation state of the body. In this report, we hope to explore the correlation between the TyG index, RF and CVD risk in the RA patient population to further improve our understanding based on previous research and to clarify whether the TyG index combined with RF has potential predictive value for CVD risk.

In this study, we found that RA patients with advanced age, low physical activity, high RF, and a high TyG index were at high risk of CVD, while "age" and "physical activity" were also independent influencing factors for CVD in the non-RA population [18]. RF is an important marker for detecting RA. High levels of RF represent a disturbed state of the autoimmune system and can activate downstream cascades of inflammatory pathways, exacerbating systemic inflammatory responses with an "immune-inflammatory" signaling pathway [20]. It has been suggested that high RF levels may be associated with the risk of CVD in patients with RA [21], and the present research further confirms this point. The TyG index is closely associated with early pathological changes in CVD, such as atherosclerosis [22], and past research has suggested that lipid levels, such as TG levels, tend to be higher in RA patients than in the normal population [23], which may corroborate our results to some extent. We further suggest that the TyG index is an independent risk factor for CVD in the RA population. Sustained inflammatory activity can lead to an imbalance in glycolipid metabolism, which accelerates the cardiovascular damage response, and we need to consider whether downstream imbalanced metabolites are feedback involved in the immune response process, which is to be confirmed by more research from the laboratory. It should not be overlooked that we found high values of CRP and Hs-CRP in RA patients regardless of the presence of CVD, which reflects the clinical reality that systemic inflammation in RA leads to elevated inflammatory factors. In patients with RA combined with CVD, although no differences in inflammatory factor values were found in this study, it is important to emphasize the risk of a "double inflammatory attack" on the cardiovascular system.

In the RA population, the TyG index, RF and CVD risk were shown to be clearly correlated. Furthermore, we plotted the ROC of the TyG index combined with RF and further evaluated the potential predictive power of the model for CVD risk. Compared with previous research, this report introduced new indicators for evaluating glucolipid metabolism, with more relevant data and a larger sample size. From the results, the TyG index and RF can both be used individually as predictors of CVD risk in the RA population, but when the two are used in combination along with age and comorbidities, the evaluation is more effective and realistic. On this basis, the diagnostic predictive value of the model might be further improved by introducing other tests that can more specifically reflect cardiovascular pathology, such as FMD [24].

Further sensitivity analysis of the results still found a correlation between the TyG index, RF and CVD risk after adjusting for age and physical activity, but some different outcome changes occurred. The TyG index did not show a statistically significant correlation with CVD risk in the "higher age" and "low-physical activity" populations. On the one hand, we hypothesized that elderly individuals usually undergo strict CVD risk control [25] with strong management measures for metabolic markers such as glucose and lipids. On the other hand, this group may have healthier daily habits than younger patients, which contribute to maintaining a stable level of glucose and lipid metabolism. Meanwhile, in the RA patients with low physical activity, no correlation was observed between the TyG index, RF and CVD risk. We consider that the patients with low physical activity are inherently in a state of metabolic disorder [26]; some other factors that we did not observe are likely to cover the effects of the predictor variables, and in addition, the lower sample size would make it difficult to obtain differences in statistics. Together, these possible causes provide an explanation for the results of the research.

The final complementary trend test performed found that RA patients with a low TyG index and RF had a lower risk of CVD, which is one of the main conclusions of our article. In normal humans, the "immune-inflammatory" response and metabolic regulation are in a synchronized and coordinated state. In RA patients, the TyG index reflects the body's glycolipid metabolic function that is disrupted by inflammatory mechanisms, and RF is involved in the whole "immune-inflammatory" response process as an important immune factor. In recent years, it has been suggested that the "immune-inflammatory" mechanism plays a key role in the pathogenesis of CVD [27] and that the bidirectional regulation of lymphocytes and antibodies can directly affect vascular and cardiomyocyte function. In the future, it may be possible to develop appropriate biologics for blood pressure and blood glucose modulation and immune-targeted therapy to prevent and control CVD risk, similar to the current targeted lipid-lowering therapies [28]. We need to further enhance our understanding of the interrelationship between RA, CVD and related immune-inflammatory factors to find more clinical evidence in the future.

## Study strengths and limitations

This study is an innovative assessment of the risk of CVD in RA patients using the TyG index and RF as the primary indicators of glucolipid metabolism and the "immune-inflammatory" response and provides some clinical evidence for a common pathology between the two types of diseases. Inevitably, there are limitations to our research, involving patients from the same hospital and almost all from the same city (Beijing, China), which may cause bias in the collection of clinical data. The research involved 418 patients with RA, and this sample size condition will still have some error, and the cohort size can be further expanded subsequently. Second, we placed more emphasis on standardized biomarkers when looking at disease activity, discarding subjective assessment methods such as the DAS-28 score. Meanwhile, anti-CCP antibody, which is thought to be more specific and predictive of disease prognosis in RA [29], did not show a significant effect in the present study, and whether this suggests that it does not participate in the immune inflammatory pathways associated with cardiovascular disease needs further discussion. In addition, a series of prospective multicenter studies can be conducted to further optimize the prediction model through long-term follow-up to evaluate the true predictive value of the TyG index and RF for the risk of CVD occurrence.

## Conclusions

In summary, we conducted a retrospective study exploring the risk associated with CVD in the RA population. The multimodal regression model demonstrated that RA patients with "high TyG index", "high RF", "advanced age", and "low-physical activity" had a higher risk of CVD, and to some extent, the TyG index combined with RF could predict the existence of CVD. Meanwhile, there was a linear trend of the TyG index and RF all with CVD risk. Based on the previous literature, this research further deepens the understanding of CVD risk prevention and control by considering RA patients as study subjects and provides clinicians with possible diagnostic and therapeutic ideas.

## Data Availability

Some, not all, original data may be shown upon request to a limited extent. However, some other data cannot be obtained due to the confidentiality of the patients’ personal information.

## Abbreviations

RA: Rheumatoid arthritis
CVD: Cardiovascular disease
CHD: Coronary heart disease
HF: Heart failure
RF: Rheumatoid factors
TyG: Triglyceride-glucose
BNP: Natriuretic peptide
Hcy: Homocysteine
TG: Triglycerides
LDL-C: Low-density lipoprotein cholesterol
Ua: Blood uric acid
Hs-CRP: High-sensitivity C-reactive protein
CRP: C-reactive protein
ESR: Erythrocyte sedimentation rate
RF: Rheumatoid factor
anti-CCP antibody: Anti-cyclic citrullinated polypeptide antibody
QTc: Corrected QT interval
LVIDd: Left ventricular end-diastolic diameter
PWTd: Left ventricular posterior wall thickness
SWTd: Septal thickness
LVMI: Left ventricular mass index
ROC: Receiver operating characteristic curve
AUC: Area under the curve
